# Supplementary material for: Incorporating functional annotation with bilevel continuous shrinkage for polygenic risk prediction
Source: BMC Bioinformatics. 2024 Feb 9;25:65. doi: 10.1186/s12859-024-05664-2 (PMC11323637; doi:10.1186/s12859-024-05664-2)
Supplement: Supplementary file 1 — Additional file 1. Supplementary Note 1. [file 12859_2024_5664_MOESM1_ESM.docx]

#### Supplementary Materials

**Supplementary Figure 1.** Illustration of the Overlapping patterns of annotation groups in the simulation studies. The $(i,j)$th element in the $K\times K$ matrix represents the intersection over union (IOU) metric for the $i$th and $j$th annotation group. Suppose $K=4$ and each annotation group ($K1$,$K2$,$K3$,$K4$) contributes to 0, 0, 10%, 90% of the total heritability respectively. Left: The non-overlapping pattern assumes the sets of genetic variants in each annotation group are mutually exclusive; Middle: Overlapping pattern I assumes a higher IOU among the annotation groups with low heritability contribution; Right: Overlapping pattern II assumes a higher IOU among the annotation groups with high heritability contribution.


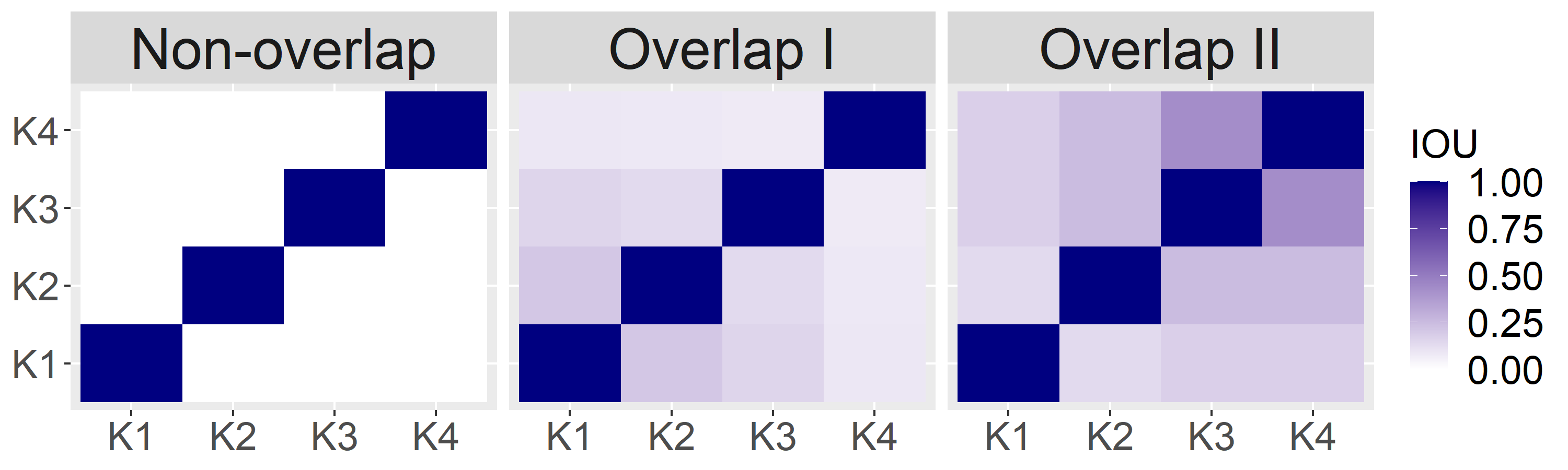


**Supplementary Figure 2.** Evaluation of $R^{2}$ from UK Biobank analysis results. Left panel: KEGG functional annotations; Right panel: Gene-based functional annotations from ANNOVAR.


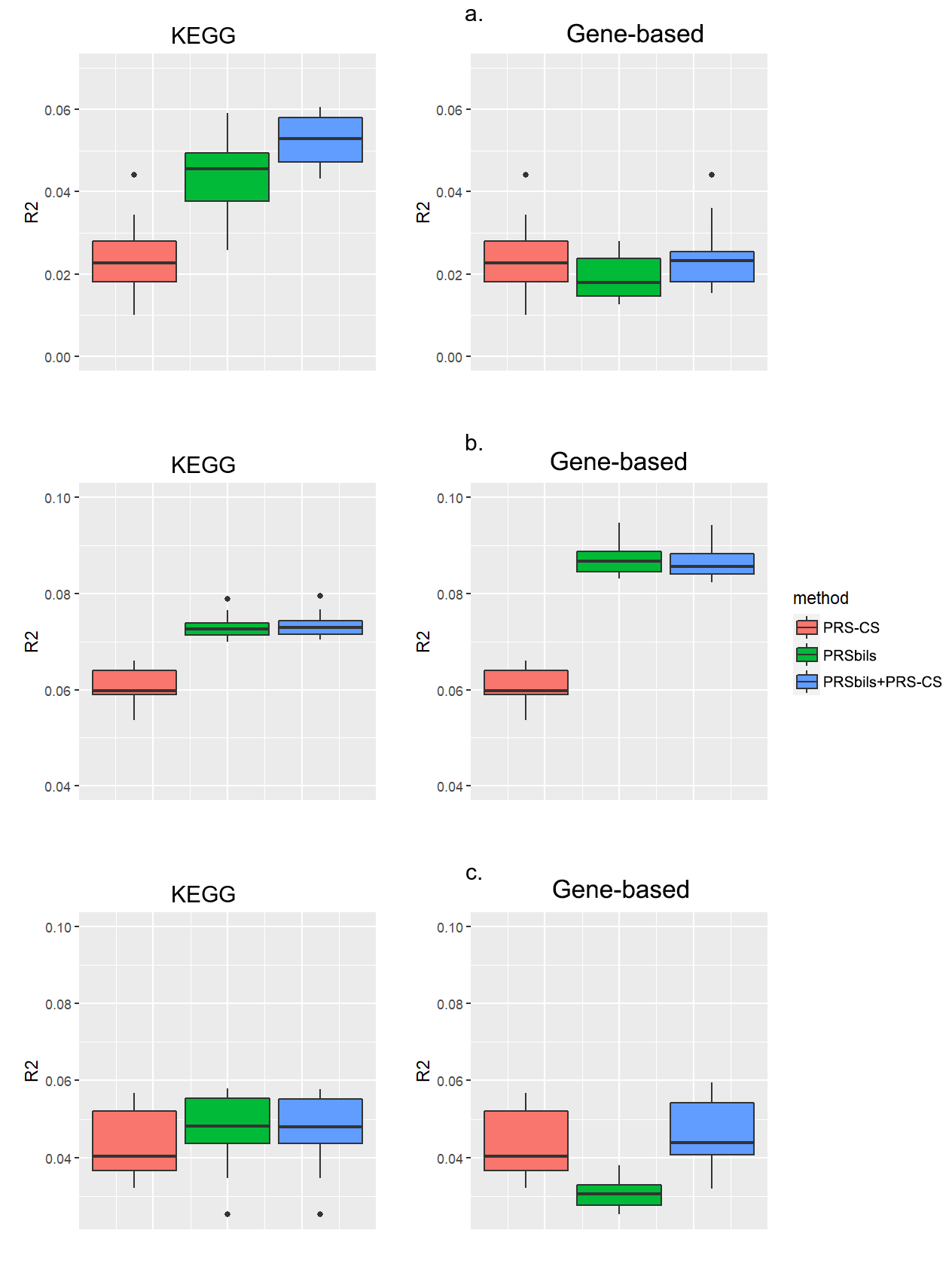


**Supplementary Figure 3.** Evaluation of $R^{2}$ from MGI data analysis results. Left panel: KEGG functional annotations; Right panel: Gene-based functional annotations from ANNOVAR. a: type II diabetes; b: BMI; c: LDL.


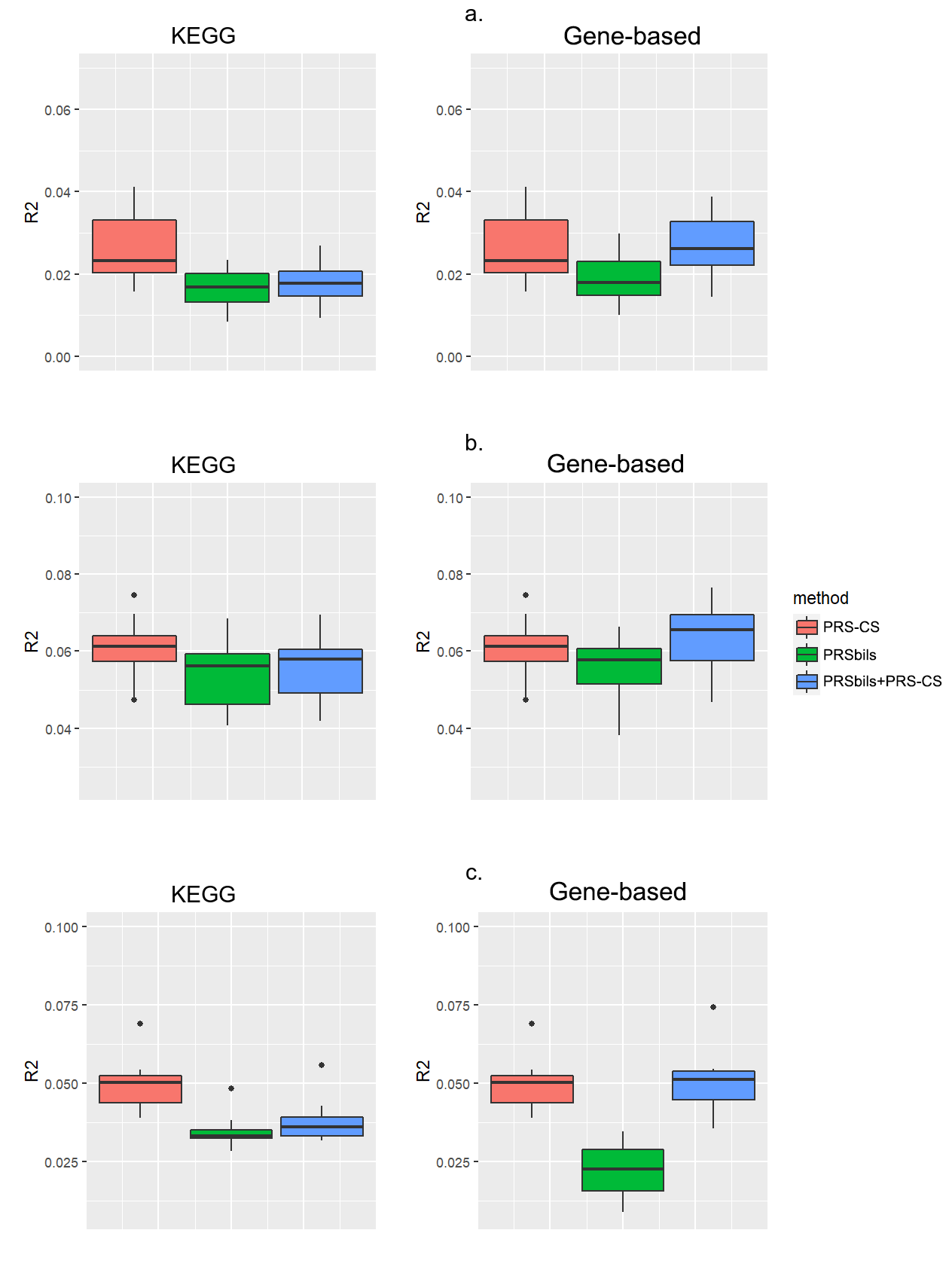


**Supplementary Figure 4.** Evaluation of KoGES analysis results. Left panel: KEGG functional annotations were used for the analysis of the proposed; Right panel: Refseq gene-based functional annotations from ANNOVAR were used for the analysis ofPRSbils. a: AUC of prediction accuracy for type II diabetes; b: Efron’s pseudo-$R^{2}$ for type II diabetes.


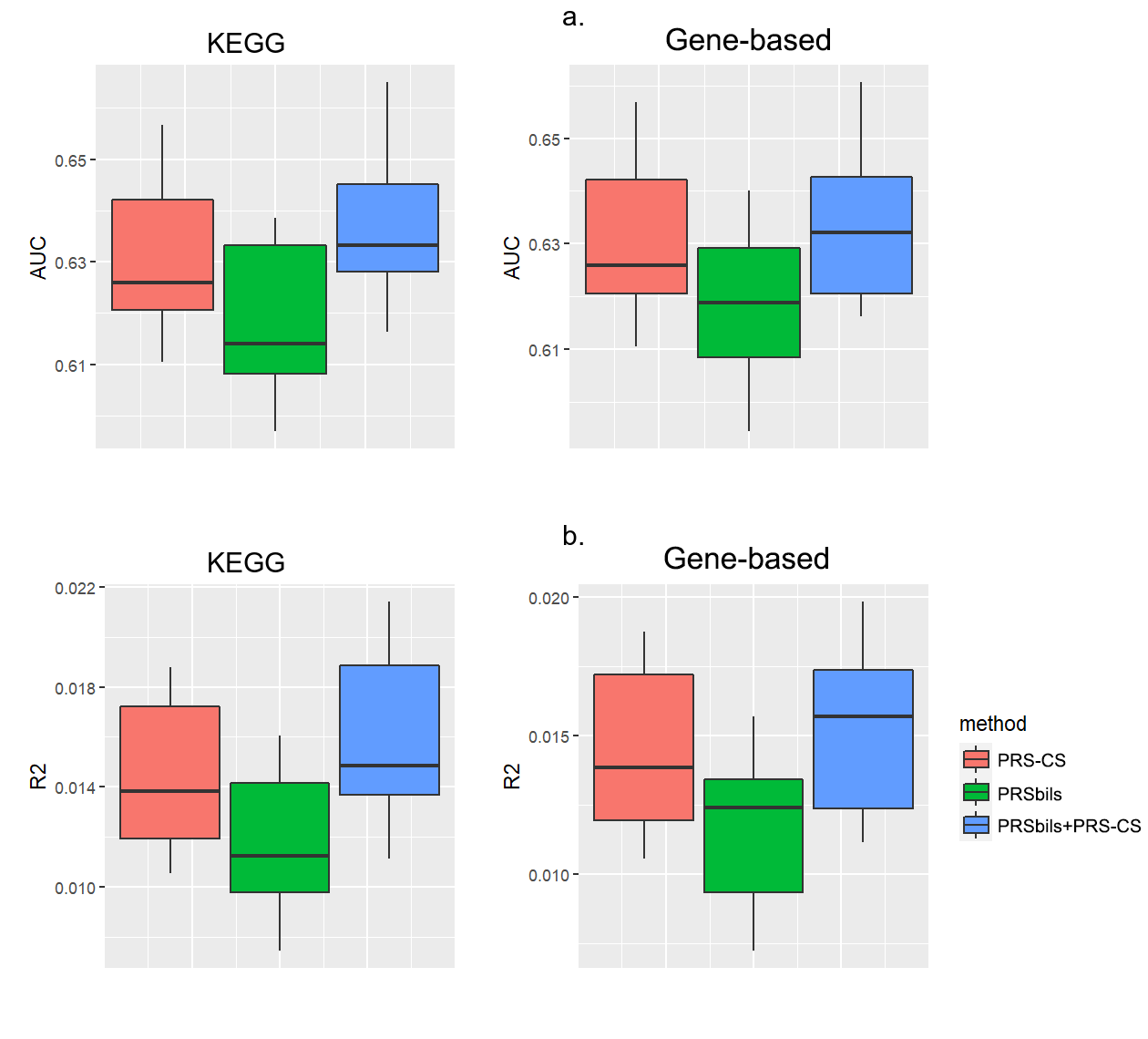


**Supplementary Figure 5.** Comparison of overall shrinkage between PRSbils (proposed) and PRS-CS (benchmark) for type II diabetes across the three data sources (UKB, MGI, KoGES). X axis: log of the absolute value of the overall shrinkage from the benchmark method; Y axis: log of the absolute value of the overall shrinkage from PRSbils. Dashed line: diagonal line indicating the same value for the two methods. a: Using KEGG annotation; b: Using Refseq gene-based annotation.


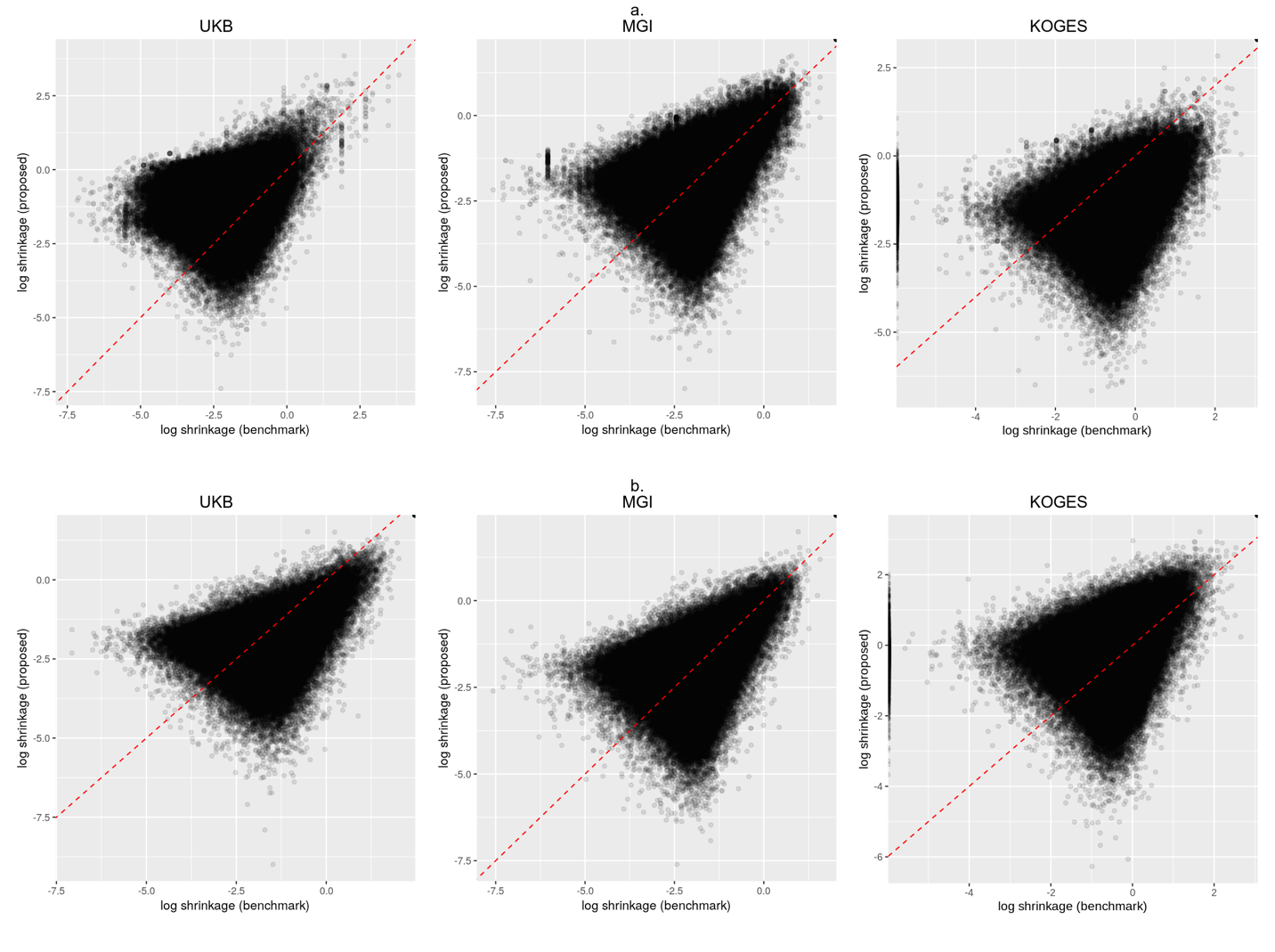


**Supplementary Figure 6.** Additional evaluation of $AUC$ from UK Biobank data analysis results for type II diabetes, with functional annotations in the baselineLD model together with KEGG annotations.


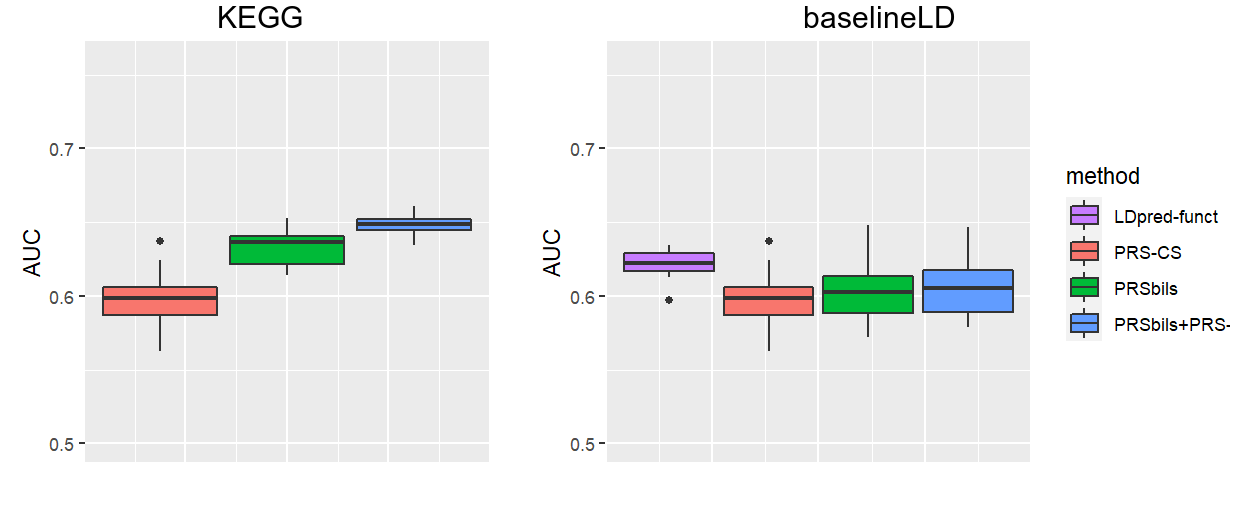


**Supplementary Figure 7.** Top 10 KEGG annotations with the highest average group-wise shrinkage from the UKB analysis for three phenotypes: type 2 diabetes, BMI, and LDL. The group-wise shrinkage for annotation group $k$ was calculated by taking the average of $\hat{\alpha}_{k}\frac{\tilde{\beta}_{j}}{\beta_{0,j}}$ for each variant $j$ within the group. $\hat{\alpha}_{k}$ is the coefficient estimate from $PRS=\sum_{k=1}^{K} \alpha_{k}PRS_{k}$, $\tilde{\beta}_{j}$ is the adjusted genetic effect from PRSbils, $\beta_{0,j}$ is the original genetic effect from the summary statistics.


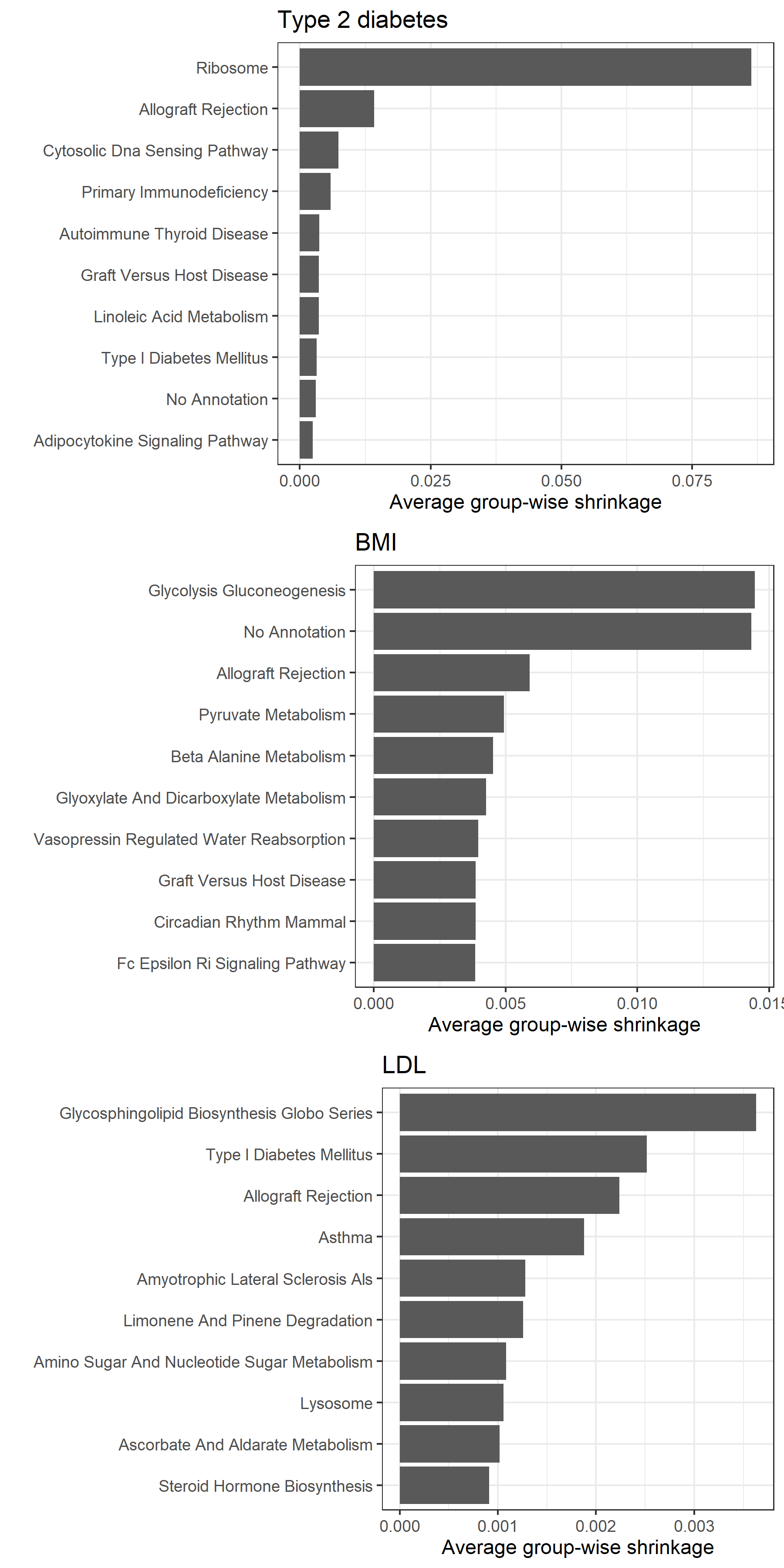


**Supplementary Figure 8.** Comparison of prediction performance with smaller sample size in simulation studies with non-overlapping annotation groups (Settings 1-5), measured by $R^{2}$. A total of M=125,000 SNPs were sampled from the UK Biobank data with 1KG as LD reference panel. Genetic effects were generated using a mixture of point-Normal models with total heritability fixed at 0.7. GWAS results from N_sumstat=50,000 simulated individuals were used as summary statistics. Prediction accuracy was evaluated in a test sample of $N_{t}est$=1,000 and 5,000 simulated individuals respectively with 10-fold cross-validation.


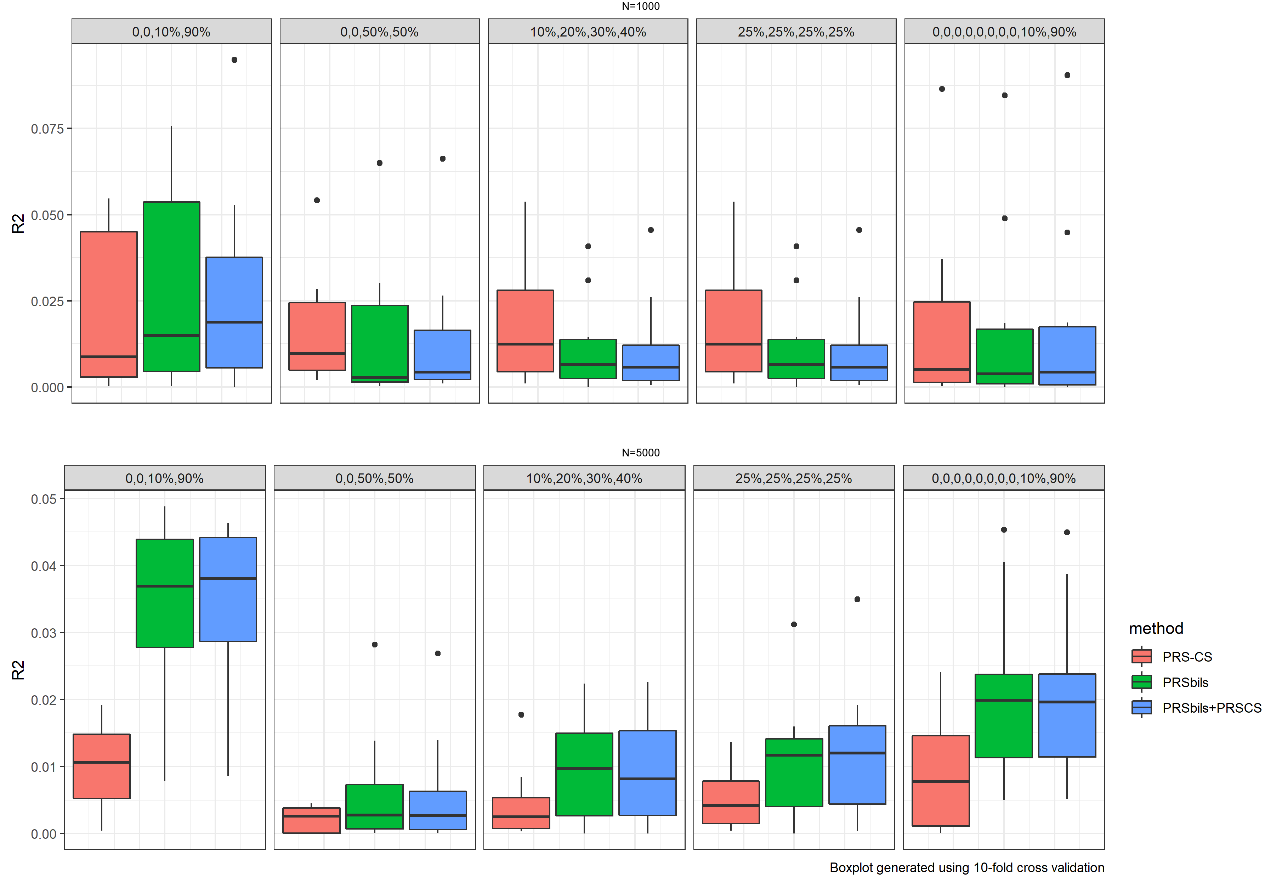


**Supplementary Figure 9.** Comparison of prediction performance with random annotation assignment in simulation studies with non-overlapping annotation groups (Settings 1-5), measured by $R^{2}$. A total of M=125,000 SNPs were sampled from the UK Biobank data with 1KG as LD reference panel. Genetic effects were generated using a mixture of point-Normal models with total heritability fixed at 0.7. After the phenotypes were generated using the “true” corresponding annotation assignment for each setting, the variants were then assigned a random “observed” annotation group with equal probability to train and test for prediction performance. GWAS results from $N_{sumstat}=50,000$ simulated individuals were used as summary statistics. Prediction accuracy was evaluated in a test sample of $N_{test}$=24,000 simulated individuals with 10-fold cross-validation.


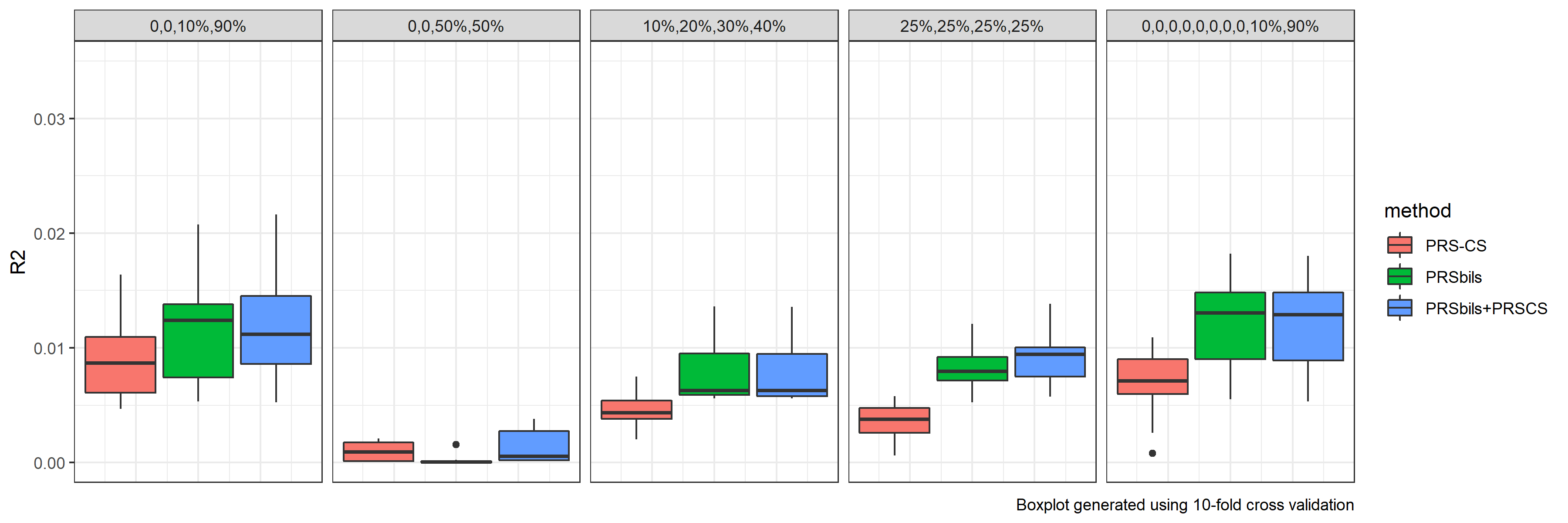


**Supplementary Figure 10.** Illustration of non-overlapping and overlapping annotations. In this illustration, the total number of variants M=4, and the total number of annotation groups K=3. (a) the non-overlapping annotation setting: under this setting, SNP1 belongs to annotation group 1 (i.e., A1=1), similarly SNP2 belongs to annotation group 1 (A2=1), and there are no overlapping variants across the three annotation groups. (b) the overlapping annotation setting: under this setting, some of the annotation groups have variants in common. For example, annotation group 1 and annotation group 2 both include SNP1. The total number of variants in this overlapping setting is $M'=\sum_{k=1}^{K} \sum_{j=1}^{M} I\left( SNP k\in A_{j} \right)$=$\sum_{k=1}^{3} \sum_{j=1}^{4} I\left( SNP k\in A_{j} \right)=5$.


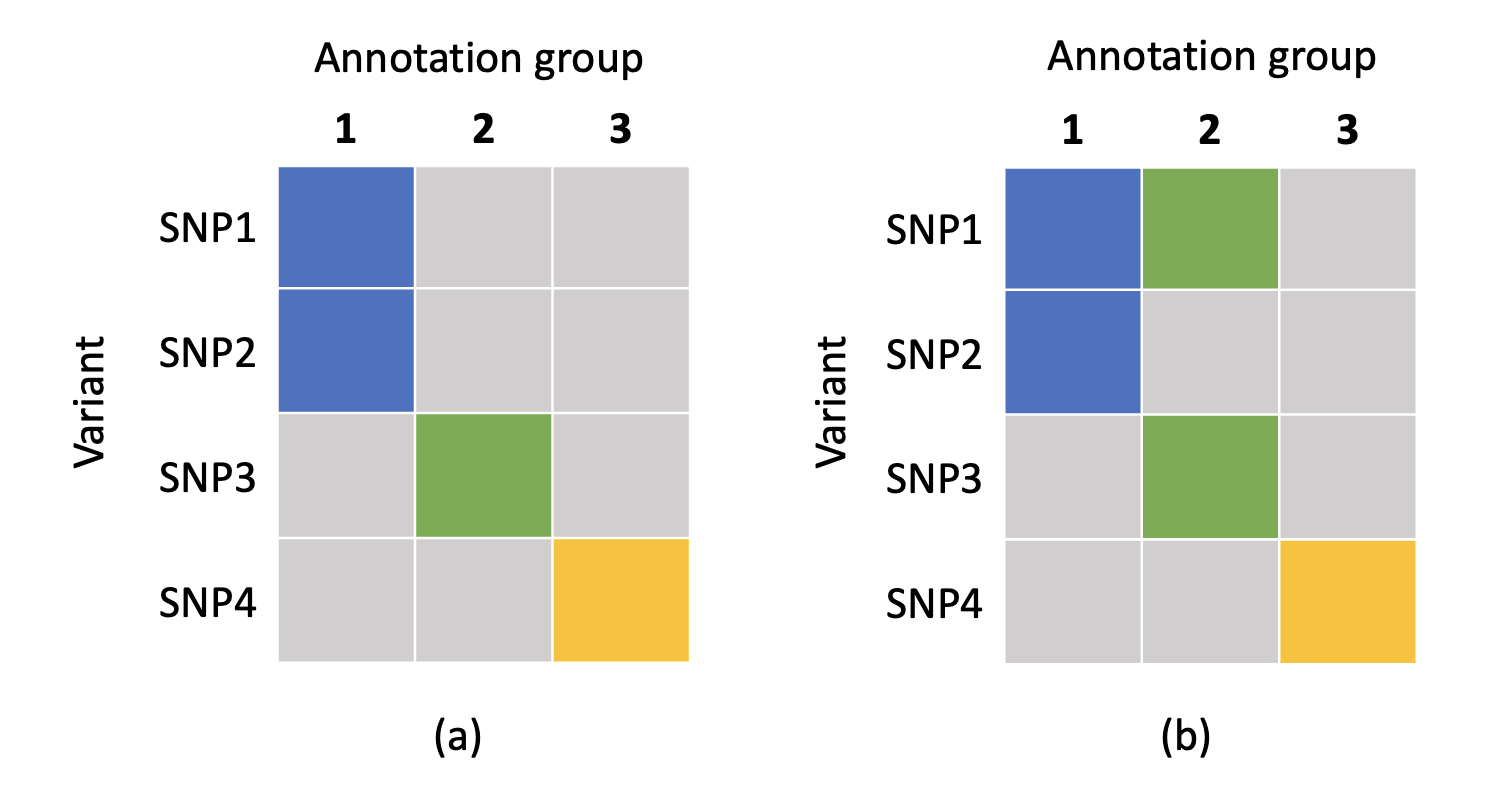


**Supplementary Table 1. A summary of data source used in the biobank data analysis. Individual level genotypes and corresponding traits were obtained from the biobanks (i.e. UK Biobank, MGI, KoGES) for validation and test sets. To build the model, we used existing GWAS analysis summaries. For Type II diabetes, we used 407,701 white British UK Biobank GWAS summaries analyzed previously for European samples (UK-Biobank Non-British White and MGI) and Biobank Japan GWAS summary for Korean samples. We also used GIANT consortium GWAS for BMI and GLGC consortium results for LDL.**

|  | **Summary statistics** | **Validation & test data (individual-level)** |
| --- | --- | --- |
| **UK Biobank analysis** | UK Biobank white British participants (type II diabetes); GIANT consortium (BMI); GLGC consortium (LDL) | UK Biobank non-British white participants |
| **MGI analysis** | UK Biobank white British participants (type II diabetes); GIANT consortium (BMI); GLGC consortium (LDL) | MGI European participants |
| **KoGES analysis** | Biobank Japan participants (type II diabetes) | KoGES participants |

#### Supplementary Note

1. Evaluation of Computation time

Computation time was evaluated using a total of 1,093,109 SNPs from 407,701 white British individuals in the UK Biobank data, with type II diabetes as phenotype. KEGG annotation was used in the PRSbils method for the evaluation. In addition to PRSbils, we also analyzed the computation time for PRS-CS and the pruning and truncating with clumping (P+T) method. PRSbils yielded a computation time of 14.0 CPU hours, compared to 12.9 CPU hours for PRS-CS and 0.5 CPU hours for P+T.

Similar to PRS-CS, PRSbils uses a Gibbs sampler to obtain the parameters in the posterior distribution iteratively, which is the major source of additional computation time compared to P+T as it involves matrix inversion with the dimension of the LD block for each iteration. While PRS-CS obtains one general set of parameters, PRSbils takes into account the annotation information and obtains one set of parameters for each annotation group separately. Therefore, when there are no overlapping annotations across the annotation groups, the computational complexity of PRSbils and PRS-CS are similar. When there are overlapping annotation groups, there would be a slight increase of computation time using PRSbils compared to PRS-CS for the additional computation on overlapping variants.
